# An Integrated Thermal Compensation System for MEMS Inertial Sensors

**DOI:** 10.3390/s140304290

**Published:** 2014-03-04

**Authors:** Sheng-Ren Chiu, Li-Tao Teng, Jen-Wei Chao, Chung-Yang Sue, Chih-Hsiou Lin, Hong-Ren Chen, Yan-Kuin Su

**Affiliations:** 1 Microsystems Technology Center, Industrial Technology Research Institute, Tainan 709, Taiwan; E-Mails: litaoteng@itri.org.tw (L.-T.T.); wayne.chao@itri.org.tw (J.-W.C.); cysue@itri.org.tw (C.-Y.S.); matthewlin@itri.org.tw (C.-H.L.); chris.chen@itri.org.tw (H.-R.C.); 2 Institute of Microelectronics and Department of Electrical Engineering, Advanced Optoelectronic Technology Center, National Cheng Kung University, Tainan 701, Taiwan; 3 Department of Electronic Engineering, Kun-Shan University, Tainan 710, Taiwan; E-Mail: yksu@mail.ncku.edu.tw

**Keywords:** MEMS, gyroscope, silicon-on-glass, temperature bias drift, delta-sigma modulation, thermal compensation

## Abstract

An active thermal compensation system for a low temperature-bias-drift (TBD) MEMS-based gyroscope is proposed in this study. First, a micro-gyroscope is fabricated by a high-aspect-ratio silicon-on-glass (SOG) process and vacuum packaged by glass frit bonding. Moreover, a drive/readout ASIC, implemented by the 0.25 μm 1P5M standard CMOS process, is designed and integrated with the gyroscope by directly wire bonding. Then, since the temperature effect is one of the critical issues in the high performance gyroscope applications, the temperature-dependent characteristics of the micro-gyroscope are discussed. Furthermore, to compensate the TBD of the micro-gyroscope, a thermal compensation system is proposed and integrated in the aforementioned ASIC to actively tune the parameters in the digital trimming mechanism, which is designed in the readout ASIC. Finally, some experimental results demonstrate that the TBD of the micro-gyroscope can be compensated effectively by the proposed compensation system.

## 1. Introduction

To identify the position and attitude in a system, inertial sensors such as gyroscopes are usually used to measure external angular rates. Optical gyroscopes are traditionally adopted because of their high accuracy, but they are bulky and expensive which makes it hard to implement them in consumer applications. For these reasons micro-machined gyroscopes have been one of most significant inertial sensor developments in the last decade owing to their small size and low cost. They have been successfully employed in many applications including tablets, smart phones, remote controls, camera stabilization, *etc.* [1,2]. The Coriolis coupling effect is the principle of the MEMS vibratory gyroscope since it identifies the angular rate input according to the detected Coriolis force acting on a vibrating mass [3]. In other words, when a vibratory gyroscope is subjected to an angular velocity, the energy is transferred from the vibrating axis to another sensing axis by the Coriolis coupling effect. The response of the sensing axis provides information about the applied angular velocity.

In driving loop control of the vibratory gyroscopes, the main target is to originate an oscillation along the vibrating axis with constant amplitude at its resonant frequency. Moreover, in order to obtain high efficiency in delivering energy from one axis to another, the resonant frequencies of both driving and sensing axes must be equal, which is also called mode-matching. However, for high-end applications such as the military, automotive, medical surgery, *etc.*, micro-gyroscopes cannot not meet the performance demands due to the bias-drift which is a critical issue for high performance micro-gyroscopes. In other words, even in the absence of any input (angular velocity) the output of the micro-gyroscope is non-zero. This offset, which is usually referred to bias-drift, is present in the measured signal. Bias-drift is a complex phenomenon which is a combination of time-, temperature- and disturbance-dependent behaviors [4]. The main consideration of the phenomenon is the effect of the temperature fluctuation of the environment. This effect is an important source error in MEMS gyroscopes and is the most discussed [5–7]. Furthermore, the relation between the temperature bias-drift and Brownian noise was investigated in the literature [8]. Some neural network-based methods were also employed in the TBD modeling and compensation of fiber optic gyroscopes and proved satisfactory [9,10]. A systematic identification and compensation method was accomplished by a JPL micro-gyroscope [11], as well as the TBD of a micro-gyroscope was investigated in terms of the resonance frequency variation induced by temperature variations. In addition, temperature dependent characteristics and compensation methods were discussed in [12]. However, the aforementioned compensation system needed an additional thermal resistor to detect the environment temperature and it was based on a MCU and PC due to the complicated compensation algorithm. In [13], the temperature characteristics of the micro-gyroscope were investigated and two different methods were proposed. However, the proposed compensation method still needed an extra temperature sensor, A/D and D/A converter, making the whole system hard to integrate on a single chip. Although a temperature control method was proposed to operate the gyroscope under optimal temperature to overcome the different temperature model due to the manufacturing errors and the influences of the peripheral circuit, an additional thermoelectric cooler and power consumption problems were induced. To eliminate the need for an extra temperature sensor, temperature self-sensing is discussed in [14]. Since the resonator frequency changes linearly with temperature, the on-chip gyroscope temperature can be measured directly. However, both the drive and detect channels are implemented on a PCB as well as the resonator frequency is detected by a PLL. In order to overcome the aforementioned need for an extra temperature sensor and system size issues, a concept design using the FPGA-based compensation method was discussed by the authors of [15] and a whole integration system is proposed in this study.

In this study, the TBD behavior of the fabricated micro-gyroscope is investigated and an active compensation system is integrated in the ASIC to suppress the undesired TBD. First, the design and fabrication principles of the micro-gyroscope are described in Section 2. Moreover, the details of the designed CMOS drive/readout circuit are addressed in Section 3. Section 4 discusses the temperature-dependent characteristics of the micro-gyroscope and deals with the proposed active thermal compensation system. Then, the behavior of the micro-gyroscope system with the proposed active thermal compensation system is verified by some experimental results and addressed in Section 5. Finally, Section 6 concludes this work.

## 2. System Architecture

**Figure 1.**
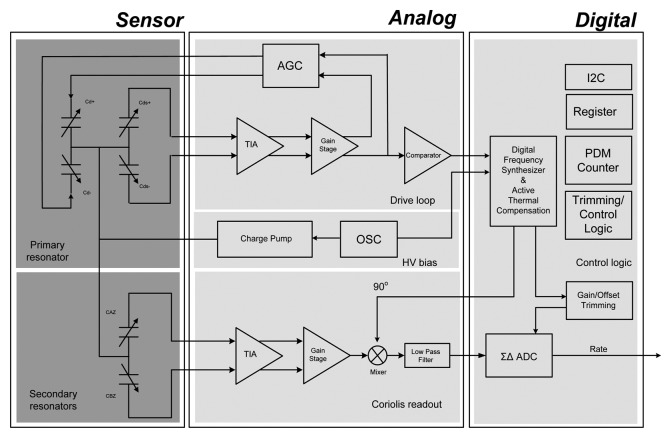
Block diagram of proposed MEMS-based gyroscope system.

The system block diagram of the proposed MEMS-based gyroscope with active thermal compensation is shown in Figure 1. The system comprises three parts: the micro-machined mechanical gyroscope, the analog part and the digital part of the ASIC. In this study, the micro-gyroscope is a vibratory type gyroscope, which consists of a resonator in the X-axis direction and a Coriolis accelerometer in the Y-axis direction. As the external angular rate about Z-axis is presented, the Coriolis accelerometer would respond to oscillations due to the resonator and is driven all the time into resonance. Moreover, the designed drive/readout ASIC consists of a driving-loop circuit to drive the resonator into resonance, a trans-impedance amplifier (TIA) to detect the Coriolis signal, as well as a gain/offset trimming ADC to adjust this output, a charge pump to supply high DC voltage for polarization and frequency adjustment, and the digital signal processing circuit for digital frequency synthesis and I2C interface communication. Furthermore, the proposed active thermal compensation system is integrated in the digital part of the ASIC to compensate the bias-drift due to the varying temperature.

### 2.1. Dynamics of MEMS Vibratory Gyroscope

**Figure 2.**
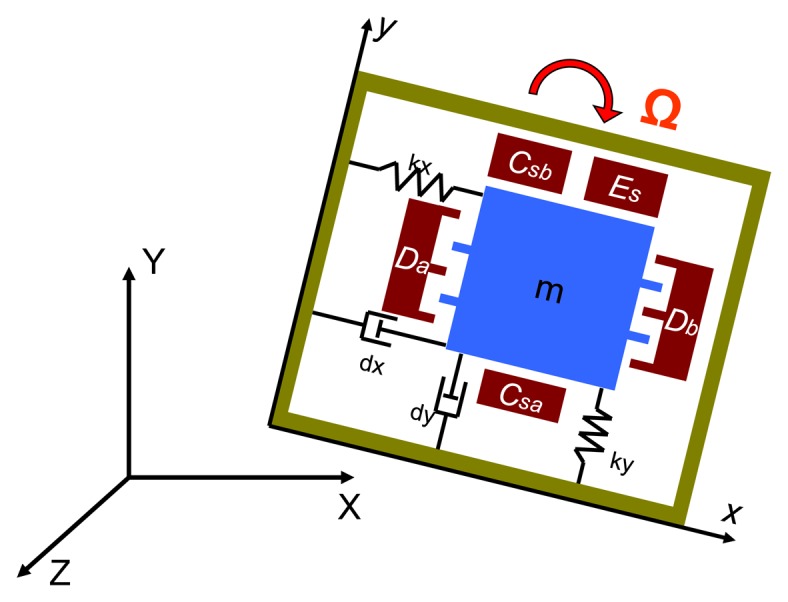
Equivalent 2-DOF mass-damper-spring system.

The equivalent 2-DOF mass-damper-spring system shown in Figure 2 is used to describe the dynamic behavior of the MEMS vibratory gyroscope. The resonator and Croiolis accelerometer are driven to vibrate about the X-axis for the drive mode. The sense mode of the Coriolis accelerometer is responded to vibrate about the Y-axis if the exerted angular rate about the Z-axis is presented.

For a constant angular rate input, assuming the linear accelerations are negligible and the frequency of the exerted angular rate is much smaller than the natural frequency of resonator and Coriolis accelerometer, the 2-DOF equations of motion of a vibratory gyroscope can be expressed as:

(1) $$m_{x}\ddot{x}+c_{x}\dot{x}+k_{x}x=\tau_{x}$$

(2) $$m_{y}\ddot{y}+c_{y}\dot{y}+k_{y}y=\tau_{y}-2m_{y}\Omega_{z}\dot{x}$$

where *x* and *y* are the coordinates of the proof mass, *m_x_* and *m_y_* are the equivalent mass related to drive mode and sense mode, *c_x_* and *c_y_* are damping coefficients, *k_x_* and *k_y_* are spring constants, *τ_x_* and *τ_y_* are the control force for drive and sense mode, the term 2*m_y_*Ω*_z_ẋ* is the Coriolis effect. Since, the variable area comb-finger capacitor is used to resonator drive the resonator, the *τ_x_* can be expressed by the following expression:

(3) $$\tau_{x}=-\nabla W=\frac{1}{2}\nabla CV^{2}=\frac{1}{2}\frac{\partial C}{\partial l_{o}}V^{2}=\varepsilon_{0}\frac{h}{g_{d}}V^{2}$$

(4) $$C=\frac{2\varepsilon_{0}hl_{o}}{g_{d}}$$

where *ε*_0_ = 8.854 × 10^−12^(*F*/*m*) is the permittivity constant of free space, *l_o_* is the overlap length, *g_d_* is the gap between the parallel capacitive plate, *h* is the structure thickness. *V* = *V_cm_* + *V_ac_* is the voltage applied on the driving electrode. For drive mode operation, the resonator is driven at resonance by the comb drive electrode pair. Then, the induced drive force can be expressed as follows:

(5) $$F_{x}=\frac{4N_{rd}\varepsilon_{0}h}{g_{rc}}[(V_{\text{XGHSPOS}}-V_{\text{com}})]V_{ac}$$

where *N_rd_* is the number of comb-fingers, *g_rc_* is the gap between comb-fingers, *V_XGHSPOS_* is the polarization DC voltage applied to the suspended structure, *V_ac_* is the AC voltage on the comb-drive electrode pair, *V_com_* is the common mode voltage. The maximum displacement of the resonator at resonance mode can be expressed as:

(6) $$x=\frac{Q_{x}F_{x}}{k_{x}}$$

where *Q_x_* is quality factor of drive mode. The induced current on the comb-sense finger of resonator can be described by the following:

(7) $$i_{d}=\dot{x}\frac{\partial C}{\partial x}(V_{\text{XGHSPOS}}-V_{\text{com}})$$

(8) $$\frac{\partial C}{\partial x}=\frac{2N_{rd}L_{\text{rco}}}{g_{rc}}$$

where *L_rco_* is the overlapped length of the sensing comb-fingers and *ẋ* is the velocity of resonator. The induced current is therefore transferred to the voltage by a trans-impedance amplifier. This voltage is then amplified and compared with the reference voltage in the automatic gain control (AGC) loop. The controlled voltage is feedback to the drive electrodes such that the resonator can be sustained to resonant. On the other hand, the Coriolis accelerometer would respond to oscillate about the Y-axis by the Coriolis acceleration if the angular rate about the Z-axis is present. The corresponding displacement of the Coriolis accelerometer can be obtained by substituting Equation (3) into Equation (1), which leads to:

(9) $$y_{0}=\frac{\omega_{x}}{\omega_{y}^{2}}\frac{2\Omega_{z}x_{0}}{\sqrt{{\left[1-{\left(\frac{\omega_{x}}{\omega_{y}}\right)}^{2}\right]}^{2}+{\left[\frac{\omega_{x}}{Q_{y}\omega_{y}}\right]}^{2}}}$$

where $\omega_{x}=\sqrt{k_{x}/m_{x}}$, $\omega_{y}=\sqrt{k_{y}/m_{y}}$ are the natural frequencies of the resonator and Coriolis accelerometer respectively. It is obvious to find that the maximum amplitude of Coriolis accelerometer, *γ*_max_, if the natural frequencies *ω_x_* and *ω_y_* are matched (*i.e.*, *ω_x_* = *ω_y_*).

### 2.2. Design and Fabrication of Micro-Machined Gyroscope

**Figure 3.**
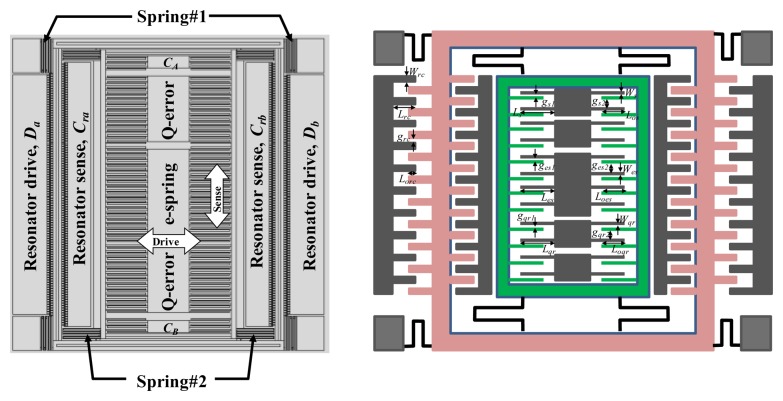
Schematic illustration of proposed 2-DOF vibratory micro-gyroscope.

The schematic illustration of the proposed 2-DOF vibratory micro-gyroscope is shown in Figure 3. It mainly consists of a vibrating resonator part and a Coriolis accelerometer part, which are arranged in outer and inner positions, respectively. The vibrating resonator part, comprises vibratory comb-drive electrodes (*D_a_* and *D_b_*), flexure springs (spring #1) and sensing capacitor set (Cra and Crb). In the Coriolis accelerometer part, flexure springs (spring #2) and sensing capacitor set (CA and CB) are designed as the angular rate sensing element to detect the responding motion induced by the Coriolis effect. In this design, a differential capacitive sensing mechanism is employed for acquiring high element sensitivity, linearity, and low temperature bias-drift. Moreover, a comb-driven electrode (e-spring) is utilized to adjust the resonant frequency of the Coriolis accelerometer. Furthermore, two comb-driven electrodes (Q-error) are designed to compensate the quadrature error, which is induced by the errora in the fabrication process of the sensing element. These comb-driven electrodes and the related error compensation will not be discussed since the proposed active thermal compensation system is the emphasis in this study. However, the research of the related quadrature error compensation can be found in the literatures [16, 17]. The detailed structure dimensions of the proposed micro-gyroscope are listed in Table 1. The natural frequency of the resonator is about 11 kHz as simulated via ANSYS.

**Figure 4.**
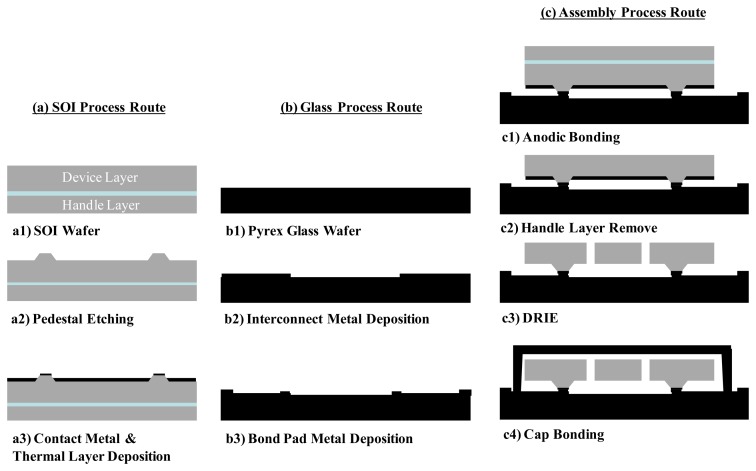
Flow of fabrication steps: (a) SOI process route, (b) Glass process route, and (c) Assembly process route.

The structure of the designed micro-gyroscope is fabricated using SOG-bulk micromachining and deep reactive ion etching (DRIE). The process flow, as shown in Figure 4, includes SOI route, glass route and assembly route. In the SOI wafer process, the pedestals are etched on the device layer first. Then, the contact metal and thermal layer are deposited. In the process of glass wafer, interconnect and bond pad metals are deposited to place interconnect/capacitor plates and bond pads separately. In the assembly process, SOI and glass wafers are assembled together by precision alignment and anodic bonding after their processes completed respectively. After the handle layer is removed, DRIE is adopted to etch the patterning structure of the device. DRIE is used in this step to etch trenches of 50 μm depth and minimum 3 μm width. This simple and robust process offers excellent control of structure thickness and desired stiction immunity during fabrication that is essential for high yield in manufacture. Finally, the fabricated gyroscope is hermetic packaged by a silicon cap in the vacuum chamber.

## 3. Drive and Readout Circuit

In order to drive the resonator and detect the signal induced by the angular rate of the vibratory micro-gyroscope, a drive/readout ASIC is designed and integrated with the fabricated micro-gyroscope by directly wire bonding. The ASIC is implemented by the 0.25 μm 1P5M standard CMOS process. Moreover, the main sub-circuits of the ASIC as shown in Figure 1 are described in detail as follows:

### 3.1. Driving-Loop Circuit

**Figure 5.**
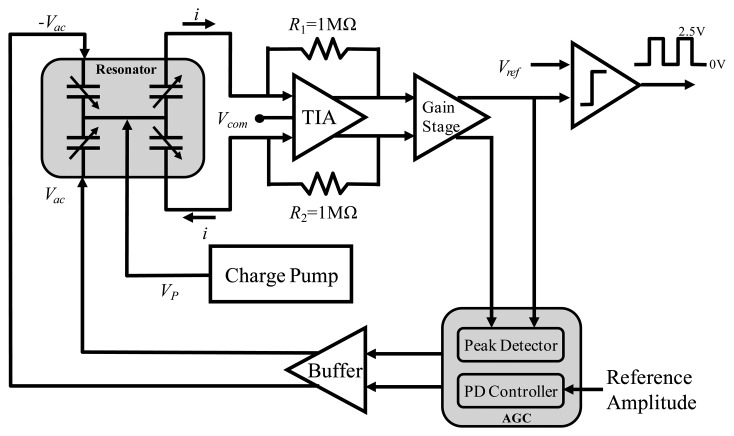
Block diagram of driving-loop for micro-gyroscope resonator.

Since the resonator is all the time driven into resonance, a driving-loop circuit is designed to self-oscillate the resonator in the micro-gyroscope. The relative block diagram of the closed-loop control for the micro-gyroscope resonator is shown in Figure 5. To detect the induced current of the driven comb fingers, a differential TIA is designed as well as a 1 MΩ feedback resistance is utilized to transform current into voltage [18]. Then, the output signal is amplified by a gain stage and feedback to the driving comb fingers for self-oscillation. Moreover, to avoid the signal saturation due to the positive feedback, an AGC circuit is adopted in the feedback path. In the AGC circuit, the peak detector detects the amplitude of the resonator output sensed by TIA is compared with the reference amplitude first. Then, a proportional differential (PD) controller is adopted to minimize the error between these signals. Finally, the anti-phase driving output of the AGC circuit is then feedback to driving comb fingers of the resonator through a buffer stage. By using the AGC circuit, the amplitude of the resonator output can be kept at same level. Besides, no extra phase shift circuit is needed since the TIA circuit can create 90 degree phase shift to compensate the resonance phase change [19].

**Figure 6.**
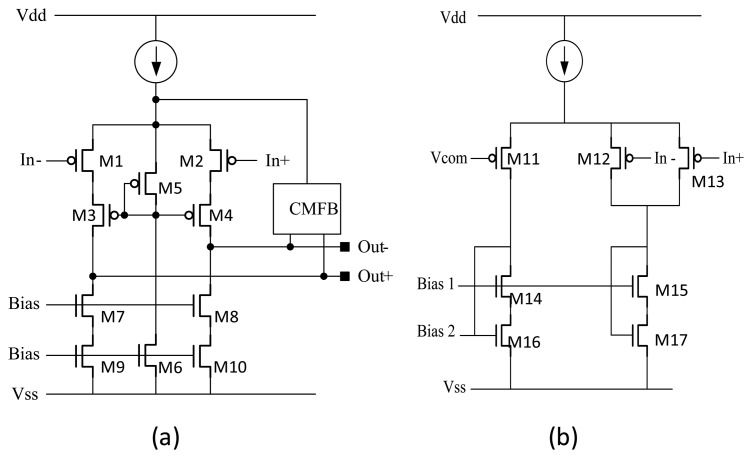
(**a**) Fully differential OPAMP used in TIA. (**b**) CMFB circuit.

On the other hand, the design of the OPAMP in the TIA circuit is important in the self-oscillated driving loop as well as the schematic is shown in Figure 6. Since the amplitude of the self-oscillated signal in drive mode is much larger than front end signal in the sense mode, the noise requirement of driving signal is less critical comparing with the Coriolis signal. Therefore, a low input noise and high gain considerations PMOS input telescopic cascade OPAMP (M1 to M10) with common-mode feedback (CMFB) (M11 to M17) is chosen in this design. Figure 6a shows the schematic of the OPAMP adopted in the TIA circuit. The designed differential-in-differential-out TIA detects the currents induced by the movement of the resonator in the drive mode and amplifies the signal with proper gain. The differential pair common-mode detector is adopted as the CMFB circuit as shown in Figure 6b. The common-mode (CM) control signal applied on the gates of M12 and M13 comes from the output of an auxiliary amplifier, which picks up the CM voltage of Out+ and Out– with two differential pairs and compares it with a reference, therefore the output common-mode voltage can be maintained automatically. The device sizes used in the OPAMP are listed in Table 2.

### 3.2. Charge Pump

**Figure 7.**
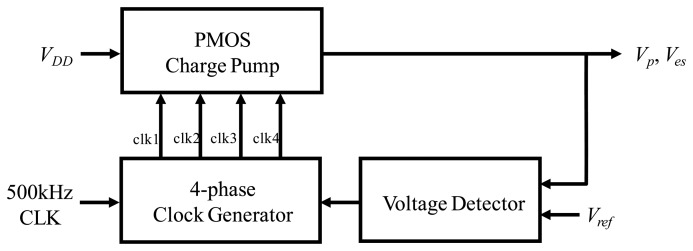
On-chip charge pump circuit.

In order to drive the resonator with lower driving voltage, a polarization voltage (Vp) is adopted in the driving-loop as shown in Figure 5. On the other hand, a frequency adjustment voltage (Ves) is applied on the elastic spring (e-spring) to tune the resonant frequency of the Coriolis accelerometer to accomplish mode-matching. Therefore, a charge pump circuit is designed in the ASIC to supply these voltages. The on-chip charge pump circuit is shown in Figure 7. In the PMOS charge pump, two auxiliary MOSFETs are introduced to update the body voltage of charge-transfer transistor, as well as the other auxiliary transistor is used to boost the gate-source voltage of the charge-transfer transistor. The charge transfer operation is realized by using two non-overlapping clock phases (clk1 and clk2) and two overlapping clock phases (clk3 and clk4) to charge the pump capacitors and to boost the gate-source voltage, respectively [20]. Therefore, high voltage supply output can be obtained by using the PMOS charge pump circuit.

### 3.3. Coriolis Readout Circuit with Gain/Offset Trimming

**Figure 8.**
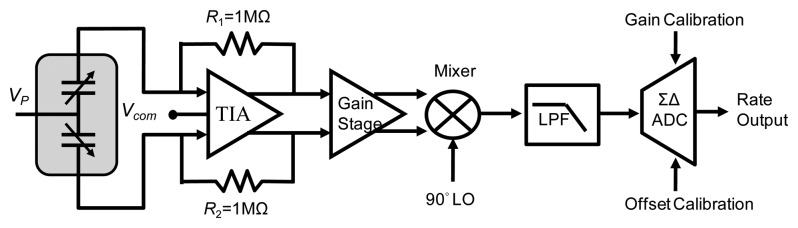
Coriolis signal readout circuit architecture.

**Figure 9.**
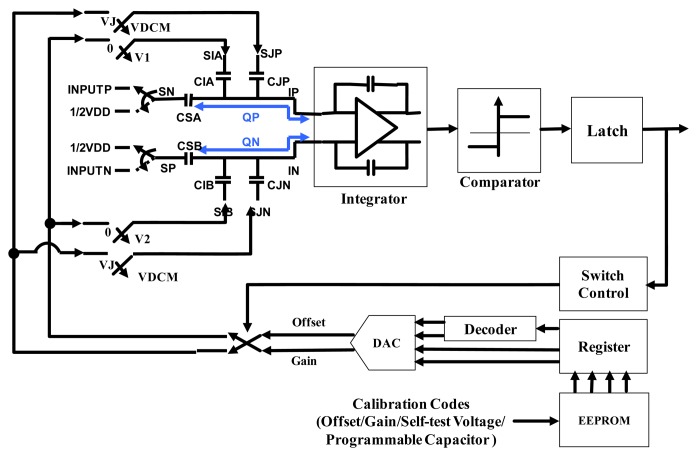
Block diagram of the delta-sigma ADC.

Figure 8 shows the proposed Coriolis signal readout circuit architecture with a gain/offset trimming function. To detect the capacitance variation of the vibratory micro-gyroscope, the same TIA used in the resonator driving loop is adopted as an analogue front end (AFE) for Coriolis signal readout. Moreover, a gain stage is adapted to amplify the output signal and send to an asynchronous demodulation circuit. The output signal of the demodulator is then feed into a delta-sigma ADC. Although the micro-gyroscopes are fabricated under same process flow, there always are some variations among different sensing elements. To ensure the consistency of the output performance of the designed micro-gyroscopes, a calibration method is designed in the delta-sigma ADC to adjust the offset and sensitivity of the sensing elements. The block diagram of the delta-sigma ADC is shown in Figure 9, where CSA and CSB are the sample-and-hold capacitors, CIA and CIB are the gain trimming capacitors as well as CJP and CJN are the offset trimming capacitors. Furthermore, the inputs of voltages of CIA, CIB and CJP, CJN are continuously clocked under two clock phases, UNITY and INTEGRATE. The input of CIA and CIB are switched between the ground voltage and the calibrated voltage VDCM-VDG or VDCM+VDG depending on the comparator output. On the other hand, the input voltages of CJP and CJN are always switched between the common voltage VDCM and the calibrated voltage VDCM +VJ no matter what the comparator output is. All of the calibrated voltages are defined by the DAC output that according to the calibration codes. Furthermore, the programmable capacitor codes and the gain/offset calibration codes are determined for a particular sensing element during the calibrated operation and stored in EEPROM.

**Figure 10.**
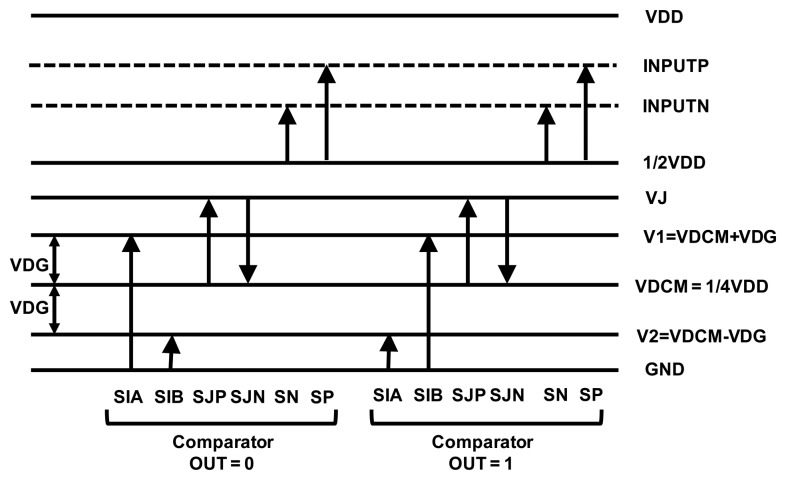
Voltage definitions of the various switch states during each clock phase.

The voltage definitions of the various switch states during each clock phase are shown in Figure 10. Moreover, the operation principle is described as follows: Suppose that comparator output is “0” such that VDCM-VDG is the input voltage for the CIB and VCOM+VDG is the input voltage for the CIA. Under this state, the input voltage SIA is switched between the two voltage level VDCM+VDG and 0 as well as the voltage SIB is switched between VDCM-VDG and 0 depending on the UNITY or INTEGRATE phase. In the UNITY phase, the charge stored on the capacitors CIA, CSA and CJP are ((VCOM-VDG)*CIA), (ΔINPUTP*CSA) and (ΔVJ*CJP) respectively. That is, the charges stored in the INPUTP of the OPAMP are:

(10) $$Q_{LP}=C_{IA}\left(V_{\text{DCM}}+V_{DG}\right)+C_{SA}\left(\Delta \text{INPUTP}\right)+C_{JP}*\Delta V_{J}$$

In the INTEGRATE phase, the charge stored on the capacitors CIB, CSB and CJN became ((VDCM-VDG)*CIB), (ΔINPUTN*CSB) and (-ΔVJ*CJN) respectively. The charges stored in the VN of the OPAMP are:

(11) $$Q_{LN}=C_{IB}\left(V_{\text{DCM}}-V_{DG}\right)+C_{SB}\left(\Delta \text{INPUTN}\right)+C_{JN}*\left(-\Delta V_{J}\right)$$

In the transition from UNITY phase to INTEGRATE phase, charge must be redistributed and the difference is accumulated on the switched capacitor integrator shown in Figure 9. Therefore, the charge packet *Q_L_* between OPAMP input VP and VN can be obtained as:

(12) $$\begin{array}{ll} Q_{L} & =Q_{LP}-Q_{LN}\\ & =\left(C_{IA}-C_{IB}\right)*V_{\text{DCM}}+\left(C_{IA}+C_{IB}\right)*V_{DG}+C_{SA}*\Delta \text{INPUTP}-C_{SB}*\Delta \text{INPUTN}+\left(C_{JP}+C_{JN}\right)*\Delta V_{J} \end{array}$$

In the same way, the charge packet *Q_H_* while comparator output is “1” can be evaluated as:

(13) $$Q_{H}=\left(C_{IA}-C_{IB}\right)*V_{\text{DCM}}-\left(C_{IA}+C_{IB}\right)*V_{DG}+C_{SA}*\Delta \text{INPUTP}-C_{SB}*\Delta \text{INPUTN}+\left(C_{JP}+C_{JN}\right)*\Delta V_{J}$$

The gain/offset trimming function of the ADC is defined as the balance of the charge, in another word, how many *Q_L_* packages are needed to balance a *Q_H_* package. Assuming that each *Q_H_* charge package is balanced by n*Q_L_ packages after the period CNT, which is equal to the period (1 + *n*)*T_CLK_. The following expression can be obtained:

(14) $$Q_{H}+n\cdot Q_{L}=0$$

(15) $$T_{\text{CNT}}=\left(1+n\right)\cdot T_{\text{CLK}}$$

Substitute Equation (14) into (15), then the count frequency *F_CNT_* is obtained:

(16) $$F_{\text{CNT}}=F_{\text{CNK}}\cdot \frac{1}{1-\frac{Q_{H}}{Q_{L}}}$$

where the fractional pulse density (FPD) output of the circuit is defined as the count frequency *F_CNT_* divided by the clock frequency *F_CLK_* as follows:

(17) $$\text{FPD}=\frac{F_{\text{CNT}}}{F_{\text{CLK}}}=\frac{1}{1-\frac{Q_{H}}{Q_{L}}}=\frac{Q_{L}}{\left(Q_{L}-Q_{H}\right)}$$

Inserting Equations (12) and (13) into (17) and set VDCM = 1/4VDD, the trimming function of the ADC can be obtained as follows:

(18) $$\begin{array}{ll} \text{FPD} & =\left(\frac{C_{IA}-C_{IB}}{C_{IA}+C_{IB}}\right)*\frac{C_{J}}{C_{A}+C_{B}}+\frac{1}{2}+\left(\frac{C_{JP}+C_{JN}}{C_{IA}+C_{IB}}\right)*\frac{\Delta V_{J}}{2V_{DG}}\\ & +\frac{C_{SA}*\Delta \text{INPUTP}-C_{SB}*\Delta \text{INPUTN}}{2V_{DG}*\left(C_{IA}+C_{IB}\right)} \end{array}$$

Assume that CIA = CIB, CSA = CSB, CJP = CJN and ΔVIN = INPUTP–INPUTN, the equation can be rewritten and divided into gain and offset parts as:

(19) $$\text{FPD}=\frac{1}{2}+\underset{\text{offset}}{\underset{︸}{\left(\frac{\Delta V_{J}}{2V_{DG}}\times \frac{CJ}{CI}\right)}}+\underset{\text{Gain}}{\underset{︸}{\left(\frac{\Delta \text{VIN}}{4V_{DG}}\times \frac{CS}{CI}\right)}}$$

where CI = CIA + CIB, CJ = CJP + CJN and CS = CSA + CSB. The gain term depends on the voltage VDG as well as the offset term depends on the voltage VDG, ΔVJ and the programmable capacitor CJ, which are used as the finest tuning codes to increase the output accuracy without increasing the DAC bits. Moreover, the output voltage of the ADC becomes:

(20) $$\begin{array}{ll} V_{\text{OUT}} & =\text{VDD}*\text{FPD}\\ & =\text{VDD}*\left(\frac{1}{2}+\left(\frac{\Delta V_{J}}{2V_{DG}}\times \frac{CJ}{CI}\right)+\left(\frac{\Delta \text{VIN}}{4V_{DG}}\times \frac{CS}{CI}\right)\right) \end{array}$$

### 3.4. Digital Signal Processing Circuit

**Figure 11.**
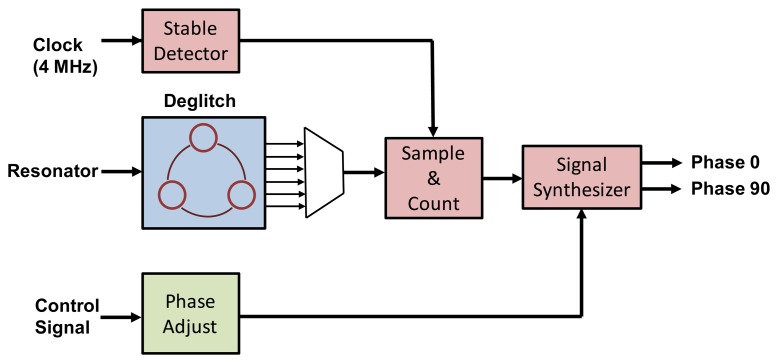
Block diagram of frequency synthesizer.

The major functions in digital circuit are frequency synthesizer and I2C interface. In the proposed vibratory micro-gyroscope, the low frequency angular rate signal is carried by the resonator's resonant frequency. In order to demodulate this rate signal and suppress the quadrature error by the feedback capacitors, a frequency synthesizer with noise suppression is utilized to generate the 90 degree and 0 degree phase delay signal, respectively. The block diagram of the frequency synthesizer circuit is shown in Figure 11. When the ASIC is power on, the stable detector is utilized to ensure the clock signal from analog part is stable for sample and count. Moreover, a comparator is adopted in driving loop to transfer the resonator signal to digital clock signal for frequency synthesis. However, the glitch is occurred in the output of the comparator due to the noise in resonator signal. Therefore, a deglitch circuit is adopted to eliminate the glitch in resonator signal. Then, the sample and count block is used to detect the frequency of resonator. Finally, the frequency synthesizer can generate the 0 degree and 90 degree phase delay signals with 50% duty cycle according to the sample and count block. Furthermore, the access in the register including the calibration codes, the active thermal compensation codes and the data transmission is implemented by the I2C interface.

## 4. Temperature Effects and Compensation Method on MEMS Gyroscope

To compensate the TBD of the micro-gyroscope, the temperature-dependent characteristic of the fabricated micro-gyroscope is investigated first. Then, according to the trimming strategies designed in the proposed delta-sigma readout circuit, the term Δ*V_J_* shown in Equation (19) can be utilized to compensate the bias-drift induced by the temperature variation of the micro-gyroscope. Therefore, an active thermal compensation system is proposed and integrated in the ASIC to adapt the calibration code in this study. Moreover, a temperature estimation method is also implemented to detect the environmental temperature without using an additional temperature sensor. The detailed descriptions of the TBD and its compensation on MEMS gyroscope are expressed as follows:

### 4.1. Temperature Effects on MEMS Gyroscope

The temperature-dependent output characteristics of MEMS gyroscope such as bias and sensitivity are the most critical issues for high performance MEMS gyroscope. Considering the drive mode operation, the micro-gyroscope is driven to resonant at constant amplitude by AGC loop and the motion of drive mode can be therefore expressed as:

(21) $$x(t)=X_{0}\sin(\omega_{x}\;t+\phi )$$

where *X*_0_ is the amplitude and the *ω_x_* is the resonance frequency of drive mode. On the other hand, the time response of sense mode by the external angular rate about Z-axis leads to Equation (9). For frequency match condition, the amplitude of Coriolis accelerometer can be simplified as |*γ*_max_| = *Q_y_*·2Ω*x*_0_/*ω_y_*. It is obvious that the output of sense mode is inverse proportion to the nature frequency, *ω_y_*. However, the nature frequency of silicon-based vibration element is strongly temperature-dependent. Therefore, the temperature-dependent nature frequency can be expanded by the Taylor series at operation temperature, *T*_0_, which can be expressed as:

(22) $$f(T)=f(T_{0})+\sum_{n=1}^{\infty }TCf_{n}\times f(T_{0})\times {\left(T-T_{0}\right)}^{n}$$

where *TCf_n_* is nth order of temperature coefficient of frequency of the micro-gyroscope. Moreover, the operation temperature, *T*_0_, is 25 °C in this work. Neglecting the high-order terms of *TCf*, the first order of *TCf*_1_ can be expressed by the following:

(23) $$TCf_{1}=\frac{TCE+\alpha }{2}$$

where *TCE* and *α* are the temperature coefficient of Young's Modulus and coefficient of thermal expansion of silicon respectively. On the other hand, the frequency match condition cannot be achieved if the operation temperature of micro-gyroscope cannot be retained at constant and the vibration amplitude of sense mode could be gradually reduced. For example, the sensing capacitances of sense mode, *C_A_* and *C_B_*, can be expressed as:

(24) $$\begin{array}{ll} C_{A,B} & =\frac{N_{CA,CB}\varepsilon A}{d\pm y}=\frac{N_{CA,CB}\varepsilon A}{d\pm y}=\left(\frac{N_{CA,CB}\varepsilon A}{d}(1\mp \left(\frac{y(T)}{d}\right)\right)\\ & =C_{A,B}^{0}\pm \delta C_{A,B}(T) \end{array}$$

where *N_CA,CB_* is the number of sensing capacitor pairs of *C_A_* and *C_B_*, *A* is the overlapped area of the sense mode sensing capacitor, *ε* is the permittivity and *d* is the gap of capacitor. According to the Equation (19), it is evident to find that both the offset term and gain term are the function of the capacitance *C_A_* and *C_B_*. Therefore, the *FPD* also shows a significant temperature characteristic. It is noted that, since the capacitor pairs in the numerator are subtracted in the gain term. The influence on the offset term is more significant than the gain term. In other words, the bias-drift is the main issue in the fabricated micro-gyroscope. Therefore, in this work, a thermal compensation system is proposed in the following section to actively tune the codes Δ*V_J_* in Equation (19) such that the temperature variation induced bias-drift can be compensated.

**Figure 12.**
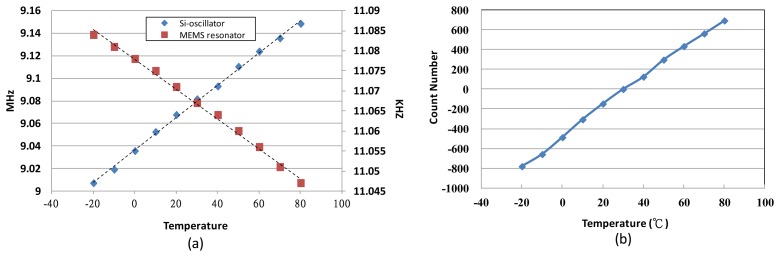
(**a**) TCfs of MEMS resonator and Si-oscillator. (**b**) Output count number difference from frequency synthesizer.

On the other hand, a modified method is adopted in this study to detect the environment temperature by the temperature characteristics of the fabricated micro-gyroscope's resonator and a silicon oscillator without using an additional temperature sensor. The temperature characteristics of the resonators are shown in Figure 12a. The *TCf*_1_ of the proposed micro-gyroscope and the silicon oscillator is about −33 ppm/°C and 157 ppm/°C, respectively. By dividing these two frequencies, a temperature dependent count number can be obtained as shown in Figure 12b. This count number is utilized to estimate the current temperature for the TBD compensation system, which is discussed in the following section.

### 4.2. Active Thermal Compensation System

Since TBD is one of the critical issues for high performance micro-gyroscopes, some compensation methods to reduce this bias-drift were investigated in previous literatures. In these literatures, an additional analog thermometer is used to detect the current temperature for TBD compensation system to generate the related compensated signals. However, the analog thermometer has some disadvantages such as low temperature resolution, large size in IC implementation and additional analog to digital converter for ASIC integration. Therefore, a digital temperature estimation method and TBD compensation system are adopted in this study since the proposed gain/offset trimming is digital-based. The current temperature is evaluated by using a second-order fitting circuit without using an additional analog thermometer. Although a conventional analog temperature sensor can be used, an additional A/D converter is needed to combine with the proposed system, which may not save silicon area. Moreover, the accuracy of analog temperature sensor depends on the resolution of the selected A/D converter, which can be improved easily by increase counting bits in the proposed system. By using the proposed TBD compensation system, the temperature effects on micro-gyroscope can be minimized by the related compensated signal according to the estimated temperature.

**Figure 13.**
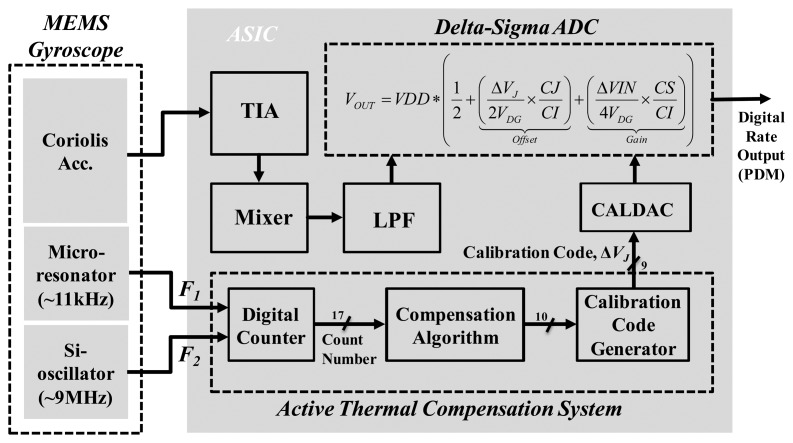
Temperature compensation system for MEMS gyroscope.

**Figure 14.**
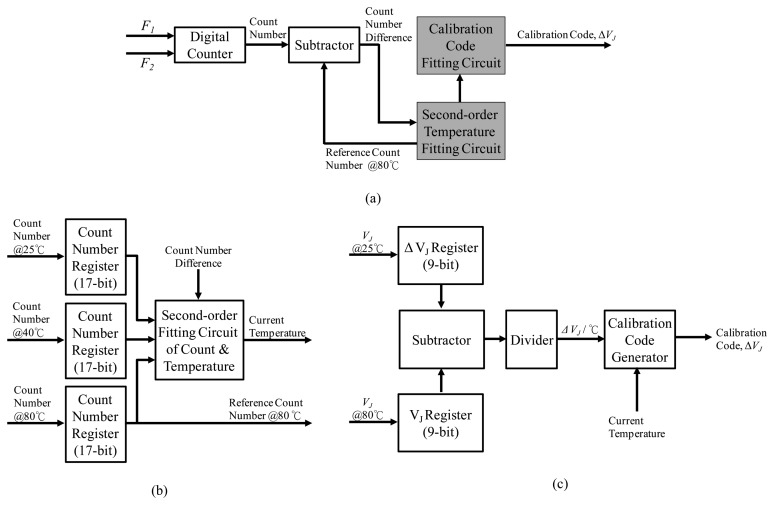
(**a**) Block diagram of proposed active thermal compensation system. (**b**) Second-order temperature fitting circuit. (**c**) Calibration code fitting circuit.

The proposed active thermal compensation system is shown in Figure 13. It consists of the MEMS gyroscope with ASIC part and the active thermal compensation system part. In the first part, the fabricated MEMS gyroscope output the resonator's resonant frequency (*F*_1_), which is about 11 kHz. Moreover, an additional silicon oscillator is implemented in the CMOS readout ASIC to generate another resonant frequency (*F*_2_), which is about 9 MHz. In the second part, the analog angular rate is obtained by the TIA, mixer and low pass filter. Then, this signal is fed to a delta-sigma ADC circuit in order to trim the gain and offset of the output rate signal. On the other hand, the proposed compensation system is implemented to adjust the calibration code Δ*V_J_*, which is represented in 9-bit digital code. This calibration code will be fed to the CALDAC circuit, which is utilized to translate the digital code into analog voltage level as shown in Figure 10. Then, the redefined voltage will be feedback to the trimming mechanism of the delta-sigma ADC circuit to adjust the *FPD* output as shown in Equation (19). The detail description of the proposed compensation system is discussed as follows: First, the input stage of the active thermal compensation system is a digital counter. It receives two frequency signals (*F*_1_ and *F*_2_), which are the output frequency of the micro-resonator and the Si-oscillator, respectively. By dividing the frequencies of the MEMS resonator and the Si-oscillator (*F*_2_/*F*_1_), a temperature-dependent count number can be obtained. Then, according to the count number, a compensation algorithm with calibration code generator is adopted to calculate a proper digital calibration code Δ*V_J_* and feedback to the delta-sigma ADC circuit by the CALDAC circuit. Finally, the output of the CALDAC redefines the voltage level in the delta-sigma ADC to adjust the digital rate output for the TBD compensation. The detail function block diagrams of the proposed active thermal compensation system are shown in Figure 14a–c and described in the following paragraph.

In Figure 14a, a digital counter is designed to count the lower frequency F1 by using the higher frequency F2 to obtain a count number first. Then, this count number is subtracted by a reference count number, which is generated by the second-order temperature fitting circuit, to obtain the difference of the count number. This count number difference is then feedback to the second-order temperature and calibration code fitting circuits to generate the calibration code. In Figure 14b, a second-order count versus temperature fitting circuit is designed to convert the input count number difference to obtain the current temperature. The fitting circuit is constructed by three pre-experimented count numbers at three preset temperatures, 25 °C and 40 °C and 80 °C, to minimize the fitting error. Moreover, in order to increase the accuracy of the fitting circuit, 17-bit register is used to store the experimented count numbers. Furthermore, the count number at 80 °C is also output to the subtractor as the reference count number. The calibration code fitting circuit is designed to fit the output calibration code *versus* temperature as shown in Figure 14c. Two pre-experimented calibration codes at two preset temperatures, 25 °C and 80 °C, are subtracted and divided to obtain the linear mapped parameter Δ*V_J_* /°C. Finally, the corresponding calibration code Δ*V_J_* under the temperature from −20 °C to 80 °C can be obtained according to the current temperature input from the second-order fitting circuit.

## 5. Experimental Results

**Figure 15.**
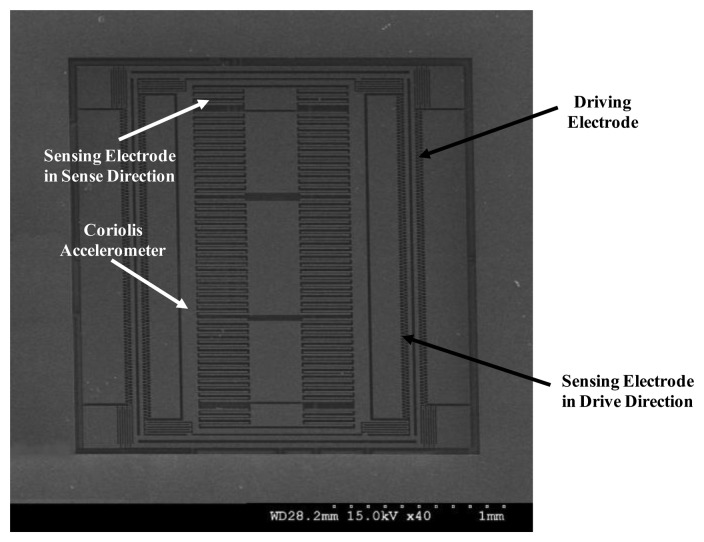
SEM photograph of fabricated micro-gyroscope.

**Figure 16.**
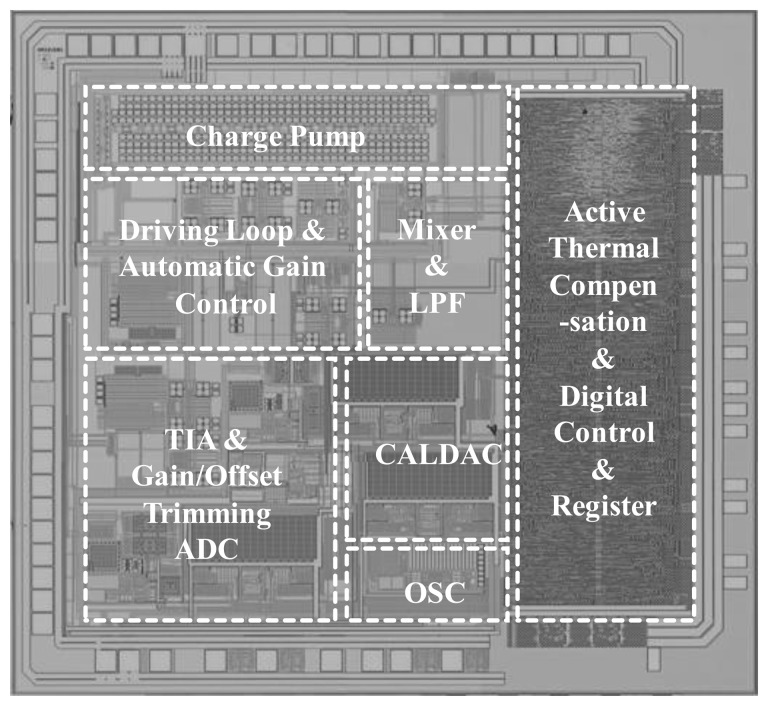
Microphotograph of the designed ASIC.

**Figure 17.**
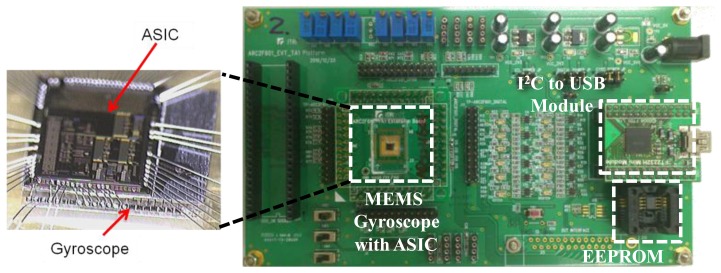
Experimental setup.

A scanning electron microscope (SEM) photograph of the fabricated micro-gyroscope is shown in Figure 15, in which the die size is 1,278 μm × 1,300 μm. Moreover, the microphotograph of the designed ASIC using 0.25 μm 1P5M CMOS standard process is shown in Figure 16, where the die size is 2,756 μm × 2,519 μm. In order to verify the function and the performance of the designed ASIC and the proposed TBD compensation system, respectively, the fabricated micro-gyroscope is integrated with the ASIC using wire bonding for the experimental setup as shown in Figure 17. Furthermore, an active compensation system is integrated in the ASIC to derive the frequency signals from the MEMS gyroscope and the embedded Si-oscillator. In addition, an USB to I2C module is adopted on the PCB for the communication between the micro-gyroscope and the PC. The calibration codes and system parameters can be written in the EEPROM by using I2C through this module.

**Figure 18.**
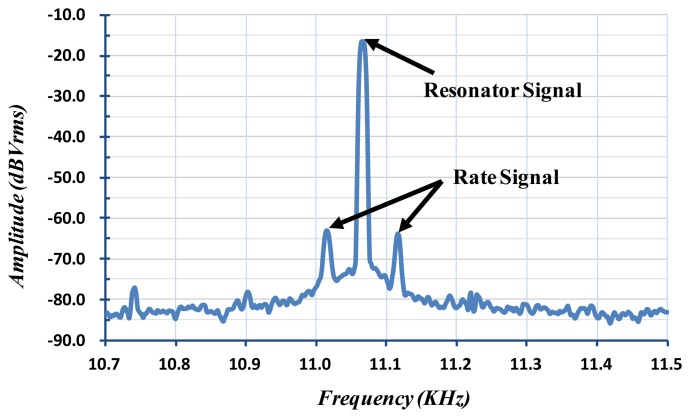
FFT spectrum and time responses of self-oscillated resonator.

**Figure 19.**
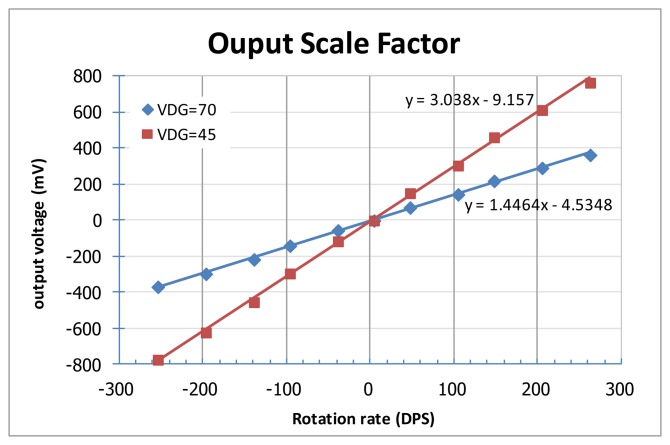
Output scale factor of micro-gyroscope.

Some experimental results of the fabricated micro-gyroscope are shown in Figures 18 and 19 to verify the function and performance. In the designed ASIC, a feedback loop and an AGC circuit are adopted as shown in Figure 5 to feedback the resonator's output signals for its self-oscillation. As the power up and white-noise applied, the resonator achieves self-oscillation by incessantly positive feedback. Moreover, the amplitude of the resonator can be controlled by the set reference voltage of the peak detector in the AGC circuit. Figure 18 shows the spectrum of the output signal when the device is subject to an 80 °/s rotation. The resonant frequency of the self-oscillated resonator of the fabricated vibratory micro-gyroscope is about 11.07 kHz. On the other hand, the resonant frequency of the Coriolis accelerometer in micro-gyroscope can also be adjusted by adjusting the supply voltage on the e-spring electrodes. By using the frequency adjustment methodology, the frequency match (*i.e.*, mode-matching) in drive-mode and sense-mode can be accomplished to obtain the high efficiency in delivering energy from the drive axis to the sense axis. Furthermore, the PCB is attached on a rate table with excited angular rate ranging from −250 to +250°/s to obtain the performance of the fabricated MEMS vibratory micro-gyroscope. The output scale factor of the vibratory micro-gyroscope from −250 to +250°/s is shown in Figure 19.

**Figure 20.**
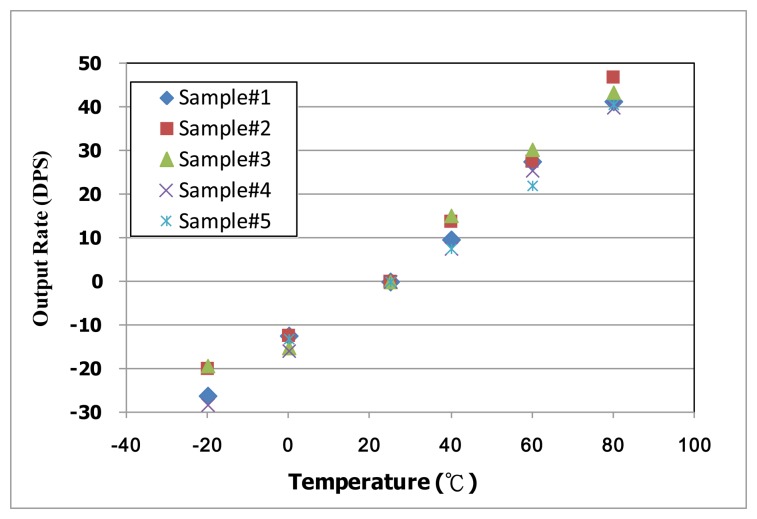
TBD of fabricated micro-gyroscope without compensation.

**Figure 21.**
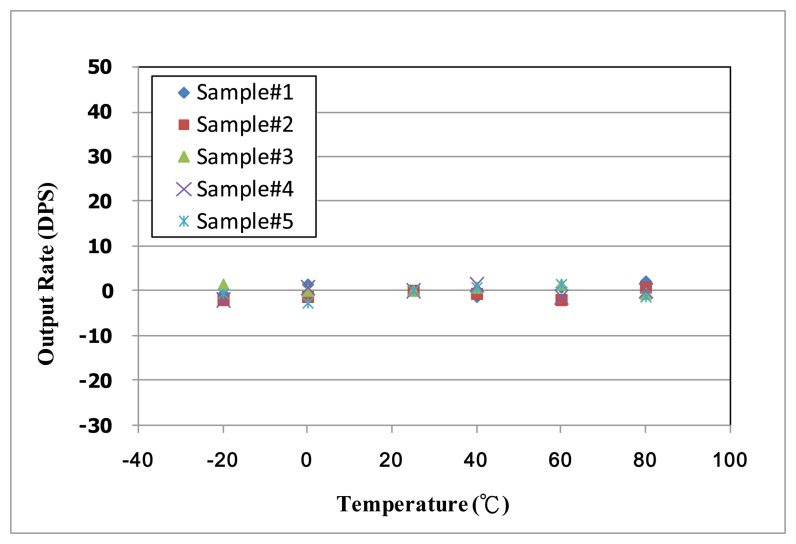
TBD of fabricated micro-gyroscope with proposed FPGA-based active thermal compensation system.

After the basic characteristics experimentation of the fabricated micro-gyroscope, the implementation of the proposed TBD compensation system is discussed. Some experimental results of the proposed active thermal compensation system are shown in Figures 20 and 21. As shown in Figure 14b, the current temperature can be estimated by the temperature dependent count number. It is noted that three preset temperatures, 25 °C and 40 °C and 80 °C, are utilized to calculate the coefficients of the second order temperature fitting circuit. On the other hand, the capability of the TBD compensation is also discussed. The output of the designed delta-sigma readout circuit can be adjusted by the calibration code Δ*V_J_*, which is adapted by the proposed active thermal compensation system. To evaluate the performance of the proposed TBD compensation system, the output signal of five fabricated micro-gyroscopes without TBD compensation are investigated and shown in Figure 20. As shown in Figure 20, the bias-drifts of these samples under temperature from −20 °C to 80 °C are about 0.63 DPS/°C. By adopting the proposed active thermal compensation system, the TBD can be compensated under ±0.03 DPS/°C as shown in Figure 21. From the experimental results, the undesired TBD can suppress effectively by using the proposed active thermal compensation system.

## 6. Conclusions and Future Work

In this work, a low temperature-bias-drift micro-gyroscope is fabricated by the MEMS process. Additionally a CMOS drive/readout circuit is designed to evaluate the performance. Moreover, the temperature-dependence characteristics of the micro-gyroscope are discussed as well as an active thermal compensation system is proposed and integrated in the designed ASIC to satisfy the demands of high performance gyroscope applications. In the proposed compensation system, the parameter in the digital trimming mechanism, which is implemented in the readout ASIC, is tuned by the calibration code such that the TBD of micro-gyroscope can be compensated effectively. On the other hand, in the conventional thermal compensation methods, an additional analog temperature sensor is placed in close proximity to the gyroscope. In this study, the environmental temperature of the micro-gyroscope is obtained directly by using frequency counting and second-order temperature fitting circuits. Therefore, the proposed active thermal compensation system is independent from the thermal lag, enabling real-time TBD compensation and eliminating additional thermometer. From the experimental results, the TBD of the micro-gyroscope can be compensated from 0.63 DPS/°C to ±0.03 DPS/°C by the proposed compensation system. In the future work, not only the offset but the gain term would be considered in the trimming function for high accuracy applications. Furthermore, an improved multi-axis compensation system will also be investigated for inertial measurement unit (IMU) applications.

## Figures and Tables

**Table 1.** Structural parameters of proposed micro-gyroscope.

**Parameter**	**Value**	**Unit**
Thickness of sensing structure, *h*	40	μm
Number of drive comb fingers of resonator, *N_rd_*	99	-
Number of sense comb fingers of resonator, *N_rs_*	108	-
Length of comb finger of resonator, *L_rc_*	15	μm
Width of comb finger of resonator, *W_rc_*	2.5	μm
Gap between comb fingers of resonator, *g_rc_*	5	μm
Overlapped length of comb finger of resonator, *L_orc_*	5	μm
Number of comb fingers of e-spring, *N_es_*	70	-
Length of comb finger of e-spring, *L_es_*	166	μm
Width of comb finger of e-spring, *W_es_*	6	μm
Small gap of comb fingers of e-spring, *g_es1_*	2.5	μm
Large gap of comb fingers of e-spring, *g_es2_*	7.5	μm
Overlapped length of comb finger of e-spring, *L_oes_*	160	μm
Number of comb fingers of Q-error, *N_qr_*	50	-
Length of comb finger of Q-error, *L_qr_*	166	μm
Width of comb finger of Q-error, *W_qr_*	6	μm
Small gap of comb fingers of Q-error, *g_qr1_*	2.5	μm
Large gap of comb fingers of Q-error, *g_qr2_*	7.5	μm
Overlapped length of comb finger of Q-error, *L_oqr_*	160	μm
Number of comb fingers of CA, *N_CA_*	15	-
Number of comb fingers of CB, *N_CB_*	13	-
Length of comb finger of CA and CB, *L_s_*	166	μm
Width of comb finger of CA and CB, *W_s_*	6	μm
Small gap of comb fingers of CA and CB, *g_s1_*	2.5	μm
Large gap of comb fingers of CA and CB, *g_s2_*	7.5	μm
Overlapped length of comb finger of CA and CB, *L_os_*	160	μm

**Table 2.** Device sizes used in OPAMP.

**OPAMP**		**Sizes**

**Components**	**Type**	**Width/Length/# of Fingers**
M1	PMOS	10 μm/2.2 μm/80
M2	PMOS	10 μm/2.2 μm/80
M3	PMOS	10 μm/1.3 μm/40
M4	PMOS	10 μm/1.3 μm/40
M5	PMOS	10 μm/2.2 μm/1
M6	NMOS	8 μm/5 μm/8
M7	NMOS	5 μm/1.3 μm/40
M8	NMOS	5 μm/1.3 μm/40
M9	NMOS	8 μm/5 μm/80
M10	NMOS	8 μm/5 μm/80
M11	PMOS	10 μm/2.2 μm/8
M12	PMOS	10 μm/2.2 μm/4
M13	PMOS	10 μm/2.2 μm/4
M14	NMOS	5 μm/1.3 μm/4
M15	NMOS	5 μm/1.3 μm/4
M16	NMOS	8 μm/5 μm/8
M17	NMOS	8 μm/5 μm/8
